# To Fry Potatoes

**Published:** 1886-01

**Authors:** 


**To Fry Potatoes.**—To fry potatoes well, slice them about an inch thick, or cut them into shavings ; dry them well in a cloth ; fry them in lard or drippings. Have the lard boiling hot and stir them until brown and crisp.

